# Transition Regret and Detransition: Meanings and Uncertainties

**DOI:** 10.1007/s10508-023-02626-2

**Published:** 2023-06-02

**Authors:** Sarah C. J. Jorgensen

**Affiliations:** Institute of Medical Science, Temerty Faculty of Medicine, University of Toronto, 1 King’s College Circle, Toronto, ON M5S 1A8 Canada

**Keywords:** Detransition, Regret, Gender dysphoria, Transgender, Gender-affirming care

## Abstract

Gender transition is undertaken to improve the well-being of people suffering from gender dysphoria. However, some have argued that the evidence supporting medical interventions for gender transition (e.g., hormonal therapies and surgery) is weak and inconclusive, and an increasing number of people have come forward recently to share their experiences of transition regret and detransition. In this essay, I discuss emerging clinical and research issues related to transition regret and detransition with the aim of arming clinicians with the latest information so they can support patients navigating the challenges of regret and detransition. I begin by describing recent changes in the epidemiology of gender dysphoria, conceptualization of transgender identification, and models of care. I then discuss the potential impact of these changes on regret and detransition; the prevalence of desistance, regret, and detransition; reasons for detransition; and medical and mental healthcare needs of detransitioners. Although recent data have shed light on a complex range of experiences that lead people to detransition, research remains very much in its infancy. Little is known about the medical and mental healthcare needs of these patients, and there is currently no guidance on best practices for clinicians involved in their care. Moreover, the term detransition can hold a wide array of possible meanings for transgender-identifying people, detransitioners, and researchers, leading to inconsistences in its usage. Moving forward, minimizing harm will require conducting robust research, challenging fundamental assumptions, scrutinizing of practice patterns, and embracing debate.

## Introduction

Gender transition is undertaken with the goal of improving the well-being of people suffering from gender dysphoria (Coleman et al., 2022). However, some have argued that the evidence supporting medical interventions for gender transition (e.g., hormonal therapies and surgery) is weak and inconclusive (Block, 2023; COHERE, 2020; Hembree et al., 2017; Ludvigsson et al., 2023; NICE, 2020a, b), and an increasing number of people have come forward over the past few years to share their experiences of transition regret and detransition (Levine, 2018b; Marchiano, 2020; r/detrans, 2023; Respaut et al., 2022; Valdes & MacKinnon, 2023). Regret is broadly defined as a negative, cognitive-based emotion involving counterfactual inference and feelings of personal agency or self-blame (Zeelenberg & Pieters, 2007). Studies evaluating regret following medical transition have used non-standardized definitions, and methods to ascertain regret have been heterogeneous (Blanchard et al., 1989; Dhejne et al., 2014; Lawrence, 2003; Narayan et al., 2021; Pfäfflin, 1993; Rehman et al., 1999; van de Grift et al., 2018; Weyers et al., 2009; Wiepjes et al., 2018). Likewise, definitions of detransition vary across studies, but most include discontinuing medications, having surgery to reverse the effects of transition, or both (Exposito-Campos, 2021; Littman, 2021; Vandenbussche, 2022). Desistance is a closely related phenomenon that is often applied to children whose gender dysphoria resolves before undergoing medical interventions (Drummond et al., 2008; Ristori & Steensma, 2016; Singh et al., 2021).

Although regret and detransition overlap in many people, they are distinct concepts and not all people who regret their transition take steps to detransition (Exposito-Campos, 2021; MacKinnon et al., 2022b). Many surgeries are irreversible, and changing or discontinuing hormonal therapies confers new risks. Likewise, not all of those who detransition feel regret about their decision to transition (Exposito-Campos, 2021; Littman, 2021; MacKinnon et al., 2022b; Pullen Sansfaçon et al., 2023; Vandenbussche, 2022). External factors, such as medical complications or discrimination, may drive some peoples’ decision to detransition (Littman, 2021; MacKinnon et al., 2022b; Turban et al., 2021b; Vandenbussche, 2022).

Detransition poses multiple medical (Biggs, 2021; Hembree et al., 2017; Irwig, 2018; Stolk et al., 2023) and bioethical (D'Angelo, 2018; Kimberly et al., 2018; Levine, 2018a) challenges. Although recent data have shed light on a complex range of experiences that lead people to detransition, research remains very much in its infancy. Little is known about the medical and mental healthcare needs of these patients, and there is currently no guidance on best practices for clinicians involved in their care. In this essay, I discuss emerging clinical and research issues related to transition regret and detransition. I begin by describing recent changes in the epidemiology of gender dysphoria, conceptualization of transgender identification, and models of care. I then discuss the potential impact of these changes on regret and detransition; the prevalence of desistance, regret, and detransition; reasons for detransition; and lastly, medical and mental healthcare needs of detransitioners. My objective is to arm clinicians with the latest information in this rapidly evolving field so they can support patients navigating the challenges of transition regret and detransition.

### The Changing Landscape of Gender Dysphoria

Gender clinics throughout the Western world are grappling with a sharp and poorly understood rise in the number of young people presenting with gender dysphoria or claiming a transgender identity (Bauer et al., 2021; Cass, 2022; Handler et al., 2019; Kaltiala et al., 2020a; Segev-Becker et al., 2020; van der Loos et al., 2023; Wood et al., 2013; Zucker, 2019). The newly presenting cases are markedly different from historical presentations (Aitken et al., 2015; Kaltiala-Heino et al., 2015; Steensma et al., 2018; van der Loos et al., 2023; Wood et al., 2013; Zucker, 2019). Before the early 2000s, most of those seeking medical interventions for gender transition presented in mid-adulthood and were almost exclusively male (Blanchard, 1989). A small number of cases seen in gender clinics were prepubescent children, often with a history of gender dysphoria beginning in early childhood and a sex ratio of two to six males to each female (Cohen-Kettenis et al., 2003; Steensma et al., 2018; Wood et al., 2013; Zucker, 2019). In youth presenting today, gender dysphoria typically begins or is first expressed in adolescence or young adulthood, and there has been a notable shift in the sex ratio to one favoring females (Aitken et al., 2015; Arnoldussen et al., 2022; Kaltiala-Heino et al., 2015; Steensma et al., 2018; van der Loos et al., 2023; Wood et al., 2013; Zucker, 2019). Moreover, significant mental health problems and neurodevelopmental comorbidities, such as autism and attention deficit disorder, frequently complicate the clinical presentation (Kaltiala-Heino et al., 2015; Thrower et al., 2020; Wood et al., 2013; Zucker, 2019).

Causes of this epidemiological shift are vigorously debated. At least three hypotheses have been proposed. First, some believe changes simply reflect society’s increased acceptance of gender diversity (Rosenthal, 2021). The magnitude of the increase in cases, coupled with the predominance of adolescent females, suggests that societal acceptance may only be part of the explanation, however. Second, others have observed the terms “transgender” and “gender-diverse” are far broader than previously used terms such as “transsexual” and encompass mere nonconformity to traditional sex stereotypes (Wright, 2022). Moreover, they point to evidence showing that, at least during childhood and adolescence, females are more likely to engage in traditionally “masculine” behaviors than males are to engage in traditionally “feminine” behaviors (Wright, 2022; Zucker et al., 1997). Last, there is evidence to support the hypothesis that epidemiological changes could be driven by prevalent maladaptive coping mechanisms together with sociocultural factors and peer influences (Haltigan et al., 2023; Littman, 2018, 2021; Withers, 2020). Many young people adopted a transgender identity in the context of family dysfunction or psychosocial issues (Bonfatto & Crasnow, 2018; D'Angelo, 2018; Kaltiala-Heino et al., 2015; Zucker, 2019). Precursors have included sexual assault and trauma (Evans, 2023; Gribble et al., 2023; Littman, 2018, 2021; Marchiano, 2021; Pullen Sansfaçon et al., 2023; Respaut et al., 2022). Parents have reported the onset of gender dysphoria in the context of heavy engagement with social media and cases have clustered within peer groups where one or multiple members identified as transgender or non-binary (Haltigan et al., 2023; Kornienko et al., 2016; Littman, 2018; Sanders et al., 2023).

### Changes in the Conceptualization of Transgender Identity and Models of Care

The marked epidemiological shift in those presenting with gender dysphoria or claiming a transgender identity has been accompanied by efforts to de-pathologize cross-sex identification, with the aim of decreasing stigma and enhancing civil rights protections for transgender people (Coleman et al., 2012; D'Angelo, 2020; Rafferty et al., 2018). In the past, it was recommended that those seeking medical interventions for gender transition undergo comprehensive psychological assessments with attempts to determine whether distress related to gender was secondary to, or better accounted for, by other mental health problems or sociocultural influences (Coleman et al., 2012; de Vries & Cohen-Kettenis, 2012). However, many transgender-identified people perceived these assessments as burdensome, intrusive, and impinging on patient autonomy (Ashley, 2019; Coleman et al., 2022). As a result of transgender advocacy, there has been a move away from what was believed to be unnecessary medical “gatekeeping” (Amengual et al., 2022; Coleman et al., 2022). Proponents of a less restrictive framework believe that transgender-identified people are best situated to determine what interventions they need to improve their well-being, regardless of age, mental health status, or duration of gender dysphoria (Ashley, 2019, 2022; Schulz, 2017). Transition-related medical interventions are now conceptualized as a means of realizing fundamental aspects of personal identity or “embodiment goals” (Ashley, 2022; Coleman et al., 2022; Schulz, 2017), in contrast to conventional medical care, which is pursued with the objective of treating an underlying illness or injury to restore health and functioning. Accordingly, in-depth mental health evaluations as a prerequisite for accessing hormonal therapy and surgery are eschewed as antithetical to “affirmation” of gender identity and are either not required or are highly abbreviated at many clinics across the USA (Ashley, 2019; Levine et al., 2022; Rafferty et al., 2018; Schulz, 2017; Terhune et al., 2022). Moreover, proponents of the gender-affirmation model argue that comorbid mental health problems should not be a barrier to accessing hormonal therapies and surgery. They attribute elevated rates of mental illness in people with gender dysphoria to prolonged exposure to hostile external responses to gender nonconformity, i.e., minority stress, which could, they believe, be alleviated by gender transition (Coleman et al., 2022; Kingsbury et al., 2022; Valentine & Shipherd, 2018). However, the minority stress model has been challenged recently by a growing number of studies that reveal high rates of mental illness and childhood adversity *pre-dating* the onset of gender-incongruent feelings (Becerra-Culqui et al., 2018; Kaltiala et al., 2020b; Kaltiala-Heino et al., 2015; Kozlowska et al., 2020; Littman, 2021). This may explain why people with preexisting mental health problems continue to struggle when social transition, hormones, or surgery fail to alleviate other problems that are frequently tied up with feelings of gender dysphoria (Kaltiala et al., 2020b; Morandini et al., 2023).

### Potential Implications of Epidemiological Changes and the Gender-Affirmation Model on Transition Regret and Detransition

The upsurge in adolescents and young adults seeking transition-related medical interventions coupled with less restrictive eligibility criteria has important implications for transition regret and detransition. First, even if only a small percentage of people who transitioned as minors or young adults later take steps to detransition, a small percentage of a population that has grown exponentially in the last decade means many more detransitioners will emerge in the coming years. Second, decisions to transition are now being made by young people who might lack the cognitive and emotional maturity to fully appreciate the long-term repercussions of their decisions. A qualitative interview study of adolescents attending two Dutch gender clinics found that most adolescents who were interviewed believed certain aspects of medical transition (e.g., loss of fertility and impact on future romantic relationships) simply cannot be understood and appreciated by young people below a certain age and most interviewees admitted they were not aware of the importance and impact of their decision to halt pubertal development at the time of their consent/assent (Vrouenraets et al., 2022). As one adolescent in the study remarked: “Of course, I was very young at the time [when I decided about starting the treatment with puberty suppression], but I had been whining about it for a long time already. It was more like: ‘I have to do it, I have to do it.’ Did I think it through [what the treatment with puberty suppression and its (possible) consequences entailed]? No.” (Vrouenraets et al., 2022).

The findings of this study are not surprising, given that the human brain does not reach maturity until the mid-twenties and the executive functions are the last abilities to fully develop (Casey et al., 2008; UNICEF, 2017). Executive functions include the ability to plan, prioritize, and strategize to achieve long-term goals as well as the ability to thoughtfully weigh information to make decisions (Casey et al., 2008; UNICEF, 2017). In the adolescent brain, connections between the prefrontal cortex or decision-making center and the amygdala or emotional center are still evolving (Casey et al., 2008; UNICEF, 2017). Consequently, adolescents often prioritize feelings over facts when making decisions (Casey et al., 2008; UNICEF, 2017). Moreover, because corticostriatal connectivity is not fully developed until adulthood, adolescents do not assess risk like adults. An adolescent’s ability to judge the value of an outcome in the future, resulting from a decision made today, is often impaired, especially when the outcome is far in the future and outside their present experience (Casey et al., 2008; UNICEF, 2017). Peer influence is another uniquely powerful factor that impacts judgement and decision-making in adolescence. Neuroimaging studies have shown that peer pressure promotes high levels of activation in the amygdala (emotional center) (Knoll et al., 2015; Somerville, 2013). This is not seen to the same extent during interactions with adults and may explain why adolescents value the opinions of their peers more than those of adults when assessing risk (Knoll et al., 2015; Somerville, 2013).

Feeling disconnected or uncomfortable with one’s body is a normal experience for adolescents and common in those with autism, anxiety disorders, eating disorders, or trauma (Bornioli et al., 2021; Krumm et al., 2017; Lantz et al., 2018). However, when distress is seen through the lens of gender identity, the priority becomes affirmation of gender identity and other important mental and/or medical healthcare needs might be overlooked or assumed to be related to gender dysphoria, i.e., diagnostic overshadowing (Cass, 2022). Moreover, a young person’s identity can be fluid and evolve over time in response to biological, psychological, and social factors (Crocetti, 2017; Kroger et al., 2009); solely relying on a subjective sense of gender identity might not be a reliable basis for medical decision-making about often irreversible interventions.

### Prevalence of Desistance, Regret, and Detransition

In the past, 61% to 98% of cases diagnosed with gender identity disorder/gender dysphoria in early childhood reconciled their gender identity with their birth sex through the natural course of puberty, if not earlier (Drummond et al., 2008; Ristori & Steensma, 2016; Singh et al., 2021). Many of these children simply grew up to be gay or lesbian adults. Traditionally “feminine” boys were far more likely to grow up to be gay men rather than transgender women, and the same was true for gender-nonconforming girls (Drummond et al., 2008; Ristori & Steensma, 2016; Singh et al., 2021). Less is known about desistance in the novel cohort of young people presenting today. Unlike in the past when clinicians actively worked with children and their parents to lessen gender dysphoria or adopted a neutral strategy of “watchful waiting” (Cohen-Kettenis & Pfäfflin, 2003; Zucker, 2008a, 2008b), many of today’s youth undergo some form of gender social transition (e.g., change in clothes, haircut, name, and pronouns; breast binding; use of opposite sex facilities, etc.) before contemplating medical interventions (Morandini et al., 2023; Olson et al., 2022; Zucker, 2020). Although social transition is often described as a neutral intervention with little, if any, long-term consequences, several studies support the hypothesis that it can concretize gender dysphoria (Olson et al., 2022; Turban et al., 2021a; Zucker, 2020). Moreover, recent evidence suggests that social transition might not be associated with improved mental health status in the short term (Morandini et al., 2023).

Nearly all minors (> 95%) who start the process of medical transition with puberty blockade at the onset of puberty, or shortly after, go on to receive cross-sex hormones (Brik et al., 2020; Carmichael et al., 2021; de Vries et al., 2011). Proponents of gender-affirming care attribute this persistence after starting puberty blockers to a robust and effective selection process, resulting in few false positives. However, this explanation is at odds with the push for abbreviated psychological assessments and a diagnostic approach, endorsed by affirming clinicians, which is based upon the premise that children “know who they are,” and adults should follow the child’s lead on decisions about medical interventions (Ehrensaft, 2016). A more troubling interpretation of the high rate of persistence following pubertal suppression is that preventing physical and sexual maturation with hormonal interventions crystalizes transgender identification (Griffin et al., 2021; Levine et al., 2022). Rather than being a “reversible pause,” puberty blockade might instead constitute the first step in a cascade of escalating medical interventions (Griffin et al., 2021).

Historical data suggest that regret following gender transition in adulthood is rare (Blanchard et al., 1989; Dhejne et al., 2014; Lawrence, 2003; Pfäfflin, 1993; Rehman et al., 1999; van de Grift et al., 2018; Weyers et al., 2009; Wiepjes et al., 2018). However, studies reporting low rates of regret are generally from an era when hormonal therapy and surgery were only undertaken under strict protocol. Regret was ascertained by a variety of methods, including retrospective review of medical charts for documentation of regret, or unvalidated questionnaires and semi-structured interviews, which are susceptible to non-response bias (Blanchard et al., 1989; Lawrence, 2003; Rehman et al., 1999; van de Grift et al., 2018; Weyers et al., 2009; Wiepjes et al., 2018). Other researchers have used a very narrow definition of regret, such as application to have birth sex reinstated as legal sex (Dhejne et al., 2014). More recently, patients with post-operative regret were identified using requests for surgical reversal, although it is unknown what proportion of those who experience regret pursue further surgery (Narayan et al., 2021). Patients who started hormonal therapies but did not proceed further with surgical removal of the ovaries or testes were often excluded from assessments of regret (Dhejne et al., 2014; Wiepjes et al., 2018); it is possible that those who were disqualified from or choose not to undergo gonadectomy had higher levels of regret than those who went on to complete their surgical transition (D'Angelo, 2018). Many studies also suffered from high rates of loss to follow-up, and patients who died by suicide or from medical complications were frequently not included in the analyses (Dhejne et al., 2014; Wiepjes et al., 2018), which may mask regret.

A Dutch study, which is often cited as demonstrating a low rate of regret in transgender adults following medical transition, illustrates how selection bias and high rates of loss to follow-up might lead to underestimates of regret (Wiepjes et al., 2018). The study included nearly 7000 patients who sought hormonal therapy and surgery from 1972 to 2015. Extensive mental health evaluations were performed on all patients to determine eligibility. In total, 70% were started on hormonal therapy and 78% of this group went on to have their ovaries or testes removed. Among those who underwent gonadectomy, rates of regret, as ascertain from retrospective review of documentation in medical charts, were only 0.3% for transgender men and 0.6% for transgender women, with an average time to regret of approximately 11 years. Regret in those who did not undergo gonadectomy was not reported and a lower proportion of patients proceeded to gonadectomy over the course of the study (79% from 1972 to 1979 versus 69% from 2005 to 2009). Moreover, 36% of patients were lost to follow-up; the prevalence of regret is unknown for those lost to follow-up.

Recent data, capturing the upsurge in the predominant adolescent-onset variant of gender dysphoria, suggest that detransition and/or regret could be more frequent than previously reported (Boyd et al., 2022; Butler et al., 2022; Cohen et al., 2023; Hall et al., 2021; Roberts et al., 2022). For example, a retrospective case-note review of 175 patients who medically transitioned at an adult gender clinic in the UK reported that 6.9% of patients detransitioned within only 16 months of starting medical transition; an additional 3.4% did not strictly meet the criteria of detransitioning but had a pattern of care suggestive of detransition (Hall et al., 2021). A further 22% disengaged from care and were discharged from the clinic without completing their planned treatment. A second study from a UK primary care practice that included 41 transgender-identifying patients (median age 22 years, range 19 to 89 years) found that 12% of those who had started hormonal treatments either detransitioned or documented regret after an average of five years (Boyd et al., 2022). In addition, 7.3% stopped hormonal therapy for medical or unknown reasons. An analysis of 1089 young people referred to pediatric endocrine clinics in England between 2008 and 2021 reported that 5.3% stopped treatment with puberty blockers or cross-sex hormones and reidentified with their birth sex before their eighteenth birthday (Butler et al., 2022). Outcomes following transfer to an adult service at age 18 were not reported.

A similar pattern has been documented in the USA. A recent study of 68 adolescents receiving care within the Children’s National gender services program found that 29% had what the authors termed a “shift” in their request for hormonal therapy over two years of follow-up (Cohen et al., 2023). Eighteen had a shift before starting hormonal therapy and two after. The most common pattern in the former group was withdrawing the request, followed by resubmitting the original request (9/18). Five youth withdrew the request with no new request at two years and one was still considering hormonal therapy as a possibility in the future. Two had a pattern of oscillating requests with one ultimately starting hormonal therapy. Among the two patients that stopped hormonal therapy, one viewed their experience as negative and the other reported meeting their gender-related goals. Another study that included 952 transgender adolescents and adults in the US military healthcare system found 29% discontinued hormonal therapies within four years (Roberts et al., 2022). While stopping hormonal therapy is not synonymous with regret, this high rate of discontinuation for a therapy that is usually intended to be life-long is notable. Moreover, regret can take up to 10 years to materialize (Dhejne et al., 2014; Wiepjes et al., 2018), so these numbers likely underestimate the full scope of regret and detransition.

### Reasons for Detransition

Recent data have shed light on a complex range of experiences that lead people to detransition (Exposito-Campos, 2021; Littman, 2021; MacKinnon et al., 2022b; Pullen Sansfaçon et al., 2023; Turban et al., 2021b; Vandenbussche, 2022). It should be noted, however, that the term detransition can hold a wide array of possible meanings for transgender people, detransitioners, and researchers, leading to inconsistences in its usage. Nonetheless, two typologies that fit under a broad definition of detransition, but that appear to have distinct causes and trajectories have emerged from the available literature (Exposito-Campos, 2021). First, external forces, such as discrimination, pressure from family, difficulties finding employment, or loss of health insurance, seem to drive the decision to detransition in studies that largely focused on participants who detransitioned at some point but later reidentified as transgender or gender-diverse (Exposito-Campos, 2021; MacKinnon et al., 2022b; Turban et al., 2021b). Moreover, rates of regret were generally low in these studies, and many participants believed their transition, detransition, and retransition were opportunities to explore and clarify their gender identity (MacKinnon et al., 2022b; Turban et al., 2021b). A second pattern is apparent in studies where most participants who detransitioned returned to identifying with their birth sex (Exposito-Campos, 2021; Littman, 2021; Vandenbussche, 2022). Participants in these studies frequently cited internal factors as the main drivers of their decision to detransition; worsening mental health or the realization that gender dysphoria was a maladaptive response to trauma, misogyny, internalized homophobia, or pressure from social media and online communities were examples of internal factors shared by participants (Exposito-Campos, 2021; Littman, 2021; Vandenbussche, 2022). Additionally, most participants in these studies deeply regretted their decision to transition and felt they were harmed by the clinicians and healthcare systems that facilitated it (Littman, 2021; Vandenbussche, 2022).

A study using data from the 2015 U.S. Trans Survey (USTS) illustrates the first narrative, whereby sociocultural forces appeared to drive the decision to detransition (Turban et al., 2021b). The USTS contains data from 27,715 transgender and gender-diverse adults recruited through LGBTQ-specific organizations, support groups, health centers, and online communities. Reasons for detransition were evaluated in the subset of 2,242 people who previously detransitioned but reidentified as transgender or gender-diverse at the time of the survey. In total, 83% cited at least one external factor as a reason for detransitioning (e.g., pressure from family members, pressure from the community, societal stigma, pressure from an employer, or difficulty finding employment, etc.); only 16% cited at least one internal factor.

Similar themes emerged from a qualitative study of 28 Canadian adults who identified as “detransitioning, retransitioning, detrans, retrans, reidentifying, experiencing a shift in gender identity after initiating transition, or having stopped transition” (MacKinnon et al., 2022b). Reasons for discontinuing or reversing gender transition included physical or mental health concerns, surgical complications, postoperative pain, unsupportive parents or romantic partners, employment discrimination, and difficulties accessing healthcare. A minority of participants experienced regret (22%) or ambivalence (11%), while most (67%) reported that they had positive feelings about the transition-related medical interventions they received. Many in the latter group identified as non-binary or gender-fluid at the time of the study and had come to accept the permanent changes to their body as part of their gender identity “journey.”

A very different narrative emerged from a study that recruited 237 participants from online communities of detransitioners who answered affirmatively to the question, “Did you transition medically and/or socially and then stopped?”(Vandenbussche, 2022). The most cited reasons for detransitioning included: realization that gender dysphoria was related to other issues (71%), health concerns (62%), transition failed to help with dysphoria (50%), found other ways to manage dysphoria (45%), unhappiness with social changes (44%), and change in political views (43%). Only 13% and 10% cited a lack of social support and discrimination as reasons for detransition, respectively. In total, 60% reported needing psychological support to cope with feelings of regret.

High rates of regret (80% any level of regret and 50% strong or very strong regret) were also reported in a study describing the experiences of 100 detransitioners recruited with outreach to sources with different perspectives about transition and detransition, including the World Professional Association for Transgender Health (WPATH) listserv and online detransition forums (Littman, 2021). All participants in this study received hormonal therapies and/or had undergone surgery and then stopped medications or had surgery to reverse changes from transition. At the time of the study, 61% of participants had returned to identifying solely as their birth sex and another 10% identified as their birth sex plus a second identification. The most frequently reported reason for detransitioning by both sexes was that their personal definition of male and female had changed so that they became more comfortable identifying with their birth sex (60%). Other reasons for detransition differed between male and female participants. Males reported ongoing mental health problems, dissatisfaction with physical results of transition, deteriorating physical health, and discrimination (36% each). By contrast, females cited concern about potential medical complications (58%), dissatisfaction with physical results (51%), ongoing mental health problems (45%), and the realization that gender dysphoria was caused by other factors such as trauma, abuse, or mental health conditions (41%). Only 17% of females cited discrimination as contributing to their decision to detransition.

### Gaps in Medical and Mental Healthcare

Gaps in the quality and accessibility of medical and mental healthcare have consistently been highlighted in studies and personal testimonies of detransitioners (Littman, 2021; MacKinnon et al., 2022b; Vandenbussche, 2022). Many detransitioners reported not feeling properly informed about health implications of treatments before undergoing them (Gribble et al., 2023; Littman, 2021; Pullen Sansfaçon et al., 2023; Vandenbussche, 2022). Likewise, many felt that they did not receive sufficient exploration of preexisting psychological and emotional problems and continued to struggle post-transition when they realized gender transition was not a panacea (Littman, 2021; Pullen Sansfaçon et al., 2023; Respaut et al., 2022; Sanders et al., 2023; Vandenbussche, 2022). Despite ongoing medical needs, most patients did not maintain contact with their gender clinic during their detransition (Littman, 2021; MacKinnon et al., 2022b; Vandenbussche, 2022). In one study, only 24% informed the physicians and clinics that facilitated their transition that they had detransitioned (Littman, 2021). For some, feelings of shame and fear of stigma were barriers to accessing medical care. Others believed the medical establishment failed them by allowing, and even encouraging, them to transition without adequate assessment or discussion of other ways to address, treat, or live with gender dysphoria (Exposito-Campos, 2021; Sanders et al., 2023). For many, needed supports and clinical expertise were simply not available (MacKinnon et al., 2022b; Vandenbussche, 2022).

Detransitioners consistently reported wanting more information about stopping or changing hormones as well as information on surgical reversal and restoration options, long-term effects of hormones, and tests to determine reproductive capacity (MacKinnon et al., 2022b; Sanders et al., 2023; Vandenbussche, 2022). Many stopped hormonal therapies “cold turkey” without medical supervision, instead turning to online detransition networks and social media (MacKinnon et al., 2022b). Patients who underwent gonadectomies as part of their transition, and therefore needed to change rather than discontinue hormonal therapy, reported that medical supervision was often suboptimal, and clinicians were not sufficiently knowledgeable to manage their care (MacKinnon et al., 2022b).

With regard to mental health needs, patients reported requiring psychological support to manage mental health problems that were unaddressed or exacerbated by transition (Marchiano, 2021; Vandenbussche, 2022). Moreover, some needed support to cope with feelings of grief and regret and to accept physical changes that could not be reversed, such as deepened voice, facial hair, and alopecia from testosterone or breast growth from estrogen (Marchiano, 2021; Pullen Sansfaçon et al., 2023; Vandenbussche, 2022). However, many therapists were reluctant to be involved in the care of detransitioners due to fears that they would be accused of performing conversion therapy if they deviated from the affirmative approach (Griffin et al., 2021). Detransitioners also described being vilified by the transgender community once they started to express doubts or questions regarding transition, compounding feelings of isolation (MacKinnon et al., 2022a; Respaut et al., 2022; Vandenbussche, 2022).

## Discussion

As outlined in this essay, there is still a great deal to learn about how to best support detransitioners. Unfortunately, gender services remain fragmented in most countries, and no one is systematically tracking how many young people regret transition or, for that matter, how many are helped by it. Moreover, many detransitioners appear to feel betrayed by the clinicians and medical system that facilitated their transition and do not return for follow-up (Littman, 2021), making assessing outcomes challenging. Given the novelty of the gender affirmation model and a “honeymoon” period of up to 10 years (Dhejne et al., 2014; Wiepjes et al., 2018), the full extent of regret and detransition in young people transitioning today, under vastly different circumstances than in the past, will not be known for many years. Moreover, regret is an emotion that is unique in its relation to personal agency (Zeelenberg & Pieters, 2007), but the exercise of personal agency in the transition process might have been limited for people who began transition as minors, whose decision-making capacity was compromised by mental illness, or who were not fully informed of known and potential adverse health implications. Feelings of profound grief about lost opportunities and negative repercussions of transition might not be fully captured by framing the emotional experience in terms of regret.

While there is growing recognition of the need to support detransitioners, clinicians lack guidance on best practices. There is no single narrative to describe their experiences and clinicians will need to be prepared to respond to the diversity of their experiences and trajectories. Some detransitioners might continue to have gender dysphoria or will have iatrogenic dysphoria related to irreversible effects of hormones and surgery and will need non-medical ways to cope with it, as well as ongoing psychological support to manage possible anxiety, regret, and shame related to detransitioning. Moreover, research is urgently needed to determine the safest way to discontinue or change hormonal therapies, as well as reversal or reconstructive surgical options.

Natural questions that arise are whether detransition can be prevented and, importantly, whether inappropriate transitions can be avoided. Multiple external forces that contribute to some peoples’ decision to detransition can be intervened upon. These include connecting patients with legal support in the face of employment discrimination and ensuring those with medical or surgical complications receive appropriate medical care (Turban et al., 2022). Comprehensive psychosocial support during the transition process could fortify people against societal or workplace discrimination, as well as pressure from friends and family (Exposito-Campos, 2021). However, clinicians should avoid creating unrealistic expectations about the challenges people will face following medical transition. As society learns to become more accepting, transgender people will still need to contend with the reality that gender identity cannot take precedence over sex in all circumstances. Moreover, when obtaining informed consent, clinicians should ensure patients understand that medical transition is not an antidote for the enduring consequences of childhood adversity, co-existing physical or phycological disadvantages, or unfortunate family circumstances (Levine et al., 2022). Additionally, failing to discuss alternatives to medicalization and promoting unsubstantiated claims that suicide is the inevitable alternative to medical transition (Biggs, 2022; Kirkup, 2020) robs patients of the opportunity to explore other management strategies.

The finding that many people were motivated to detransition due to a change in their personal definition of male or female, which in turn led to them to feel more comfortable identifying with their birth sex (Littman, 2021), raises questions about whether some individuals interpret gender nonconformity as transgender identification. Engaging in traditionally “feminine” tasks does not make one less of a man. Likewise, “masculine” pursuits are not incompatible with womanhood. Clothes, haircuts, colors, mannerisms, etc., do not have a sex. The original Dutch protocol for transition of minors emphasized the role of psychotherapy in fostering self-acceptance of gender-nonconformity prior to contemplating medical interventions (de Vries et al., 2006) and a growing body of literature describes an exploratory psychotherapeutic approach that can help people expand their definition of what it means to be a man or a woman, thereby potentially eliminating the need for transition-related medical interventions (Bonfatto & Crasnow, 2018; Churcher Clarke & Spiliadis, 2019; Evans, 2022; Hakeem, 2018).

Since our ability to predict with who will benefit from medical transition and who will be harmed is limited, it is imperative that we learn from the experiences of detransitioners to improve the process of evaluation, counseling, and informed consent. Communication and language have been important in the debate about appropriate medical care for people with gender dysphoria. The finding that many detransitioners felt the information they received about medical interventions was overly positive about potential benefits while risks were downplayed (Littman, 2021), suggests communication needs to be more balanced, evidence-informed, and precise. Patients, parents, and the general public in North America are often told that the use of hormonal therapies and surgery is uncontroversial and backed by rigorous science, yet there is a great deal of disagreement within the international medical community about the benefits and harms of these interventions (Block, 2023). Multiple European countries that were once strong proponents of the youth gender transition are now reversing course and prioritizing psychological support and treatment for comorbid psychiatric conditions after their own systematic reviews found the evidence underpinning gender-affirming medical interventions to be weak and uncertain (Block, 2023; Cass, 2022; COHERE, 2020; Socialstyrelsen, 2022).

Regarding language, efforts to normalize or destigmatize treatments for those who might benefit from them have obscured the severity of some interventions. One’s reaction to “top surgery” versus “double mastectomy,” “bottom surgery” versus “phalloplasty” or “vaginoplasty,” for example, are likely to be very different. Even the term “detransition” has become verboten among some transgender advocates due to fears that it could be weaponized to deny medical care to those seeking gender transition (Turban et al., 2022). Phrases such as “gender-identity journey” and “dynamic desires for gender-affirming medical interventions” have been proposed as alternatives (Turban et al., 2022). However, we should be cautious about adopting euphemisms that might mask iatrogenic harm.

### Conclusion

*Primum non nocere* has been the guiding tenet of medicine for millennia. Adhering to this principle is relatively straightforward when a diagnosis is based on clearly defined objective criteria and practice is guided by high-quality evidence establishing a treatment as both safe and effective. However, complex clinical cases are often fraught with diagnostic uncertainty and many treatments are not backed by compelling scientific evidence. In these situations, minimizing iatrogenic harm requires application of cautious, thoughtful clinical judgement, meticulous examination of the data that are available, as well as a willingness to change practice in the face of new evidence. Moving forward, the field of gender medicine will need to commit to conducting robust research, challenging fundamental assumptions, scrutinizing their practice patterns, and embracing debate.

## Data Availability

Not applicable.
